# Reconstructed data of landings for the artisanal beach seine fishery in the marine-coastal area of Taganga, Colombian Caribbean Sea

**DOI:** 10.1016/j.dib.2020.105604

**Published:** 2020-04-22

**Authors:** Jairo Altamar, Jesús Correa-Helbrum, Diego Restrepo-Leal, Carlos Robles-Algarín

**Affiliations:** a Universidad del Magdalena, Facultad de Ingeniería, Carrera 32 No 22 – 08, Santa Marta, Colombia; b Escuela de Ciencias del Mar, Facultad de Ciencias del Mar y Geografía Pontificia Universidad Católica de Valparaíso, Valparaíso, Chile

**Keywords:** Beach seine, *Caranx crysos*, *Euthynnus alletteratus*, dynamic artificial neural network, Colombian Caribbean

## Abstract

This paper presents a dataset on the abiotic (oceanographic, atmospheric and global climatic indices) and fishery variables of the marine-coastal area of the Magdalena Province in the area between Taganga and Bahía Concha, located north of Santa Marta in the Colombian Caribbean. The abiotic variables were downloaded from the satellites of the National Aeronautics and Space Administration (NASA) and the meteorological stations of the Institute of Hydrology, Meteorology and Environmental Studies (IDEAM). The fishery variables were obtained through field trips in the study area. A dynamic artificial neural network was implemented to reconstruct the missing data in the fishery variables from the known abiotic variables (Precipitation, North Atlantic Oscillation and Multivariate ENSO Indices). In this way, a dataset was obtained that is important to determine the historical changes of fishery resources for the study area and to make catch forecasts incorporating the variability of the environmental conditions (atmospheric and oceanographic).

Specifications table

**Table:** 

Subject	Computer science.
Specific subject area	Computer science applications, artificial intelligence.
Type of data	Table, Figures.
How data were acquired	The data of the abiotic variables were obtained from the NASA ocean color platform and the IDEAM automatic weather stations. The fishery variables were obtained through surveys applied to fishers of the study area (the form used are presented in the supplementary material). The reconstruction of the missing data was performed with a dynamic artificial neural network.
Data format	Raw, filtered and analyzed.
Parameters for data collection	An analysis was performed to establish if the data contained NaN values and outliers according to the expected behavior of the variables. In these cases, data were deleted.
Description of data collection	The images of abiotic variables (oceanographic and atmospheric) were obtained from the NASA Ocean Color platform, which uses the MODIS-Aqua (Moderate Resolution Imaging Spectroradiometer) sensor. Precipitation data were acquired from the IDEAM weather station, located at Simón Bolívar airport in Santa Marta, Colombia. The reconstructed data of the landings were obtained after processing with a dynamic artificial neural network (ANN).
Data source location	The coastal marine zone of Magdalena Province between Taganga (11 ° 16′03 "N, 74 ° 11′27" W) and Bahía Concha (11 ° 17′50.5 "N, 74 ° 08′59.8" W), located north of Santa Marta, Colombian Caribbean.
Data accessibility	Data is with this paper.

## Value of the data

• These data allow to determine historical changes in the abundance of fishing resources captured by the beach purse seine fisheries, particularly when the aim of the study are data-poor fisheries.
• The data of time series benefits the managers of the fishery resources, the scientists and mainly the users who take advantage of this fisheries (fishermen, trader and community in general). These actors could make consensual decisions to implement fisheries management measures.
• With the times series is possible to associate fishing and environmental information to generate catch forecasts that incorporate the variability of atmospheric and oceanographic conditions.
• The sustainability of fishery resources is key to the well-being of communities. Thus, the estimation of missing data and the reconstruction of the time series will allow fishery resource managers to take management measures for some overexploited species.
• Additionally, the reconstruction of the time series will allow to establish relationships between the fishing landings and the global climatic indexes.

## 1 Data

Table 1 shows a summary of the abiotic and fishery variables. The complete data for all these variables are presented in Table 1 of the supplementary material, in which the missing values are represented with NaN.

**Table 1:** Abiotic (oceanographic, atmospheric and global climatic indices) and fishery variables.

Variable	Number of missing data	Number of current data	Loss (%)
Year	0	288	0%
Month	0	288	0%
SST (Taganga)	0	288	0%
SST (Arriba aguja)	0	288	0%
SST (Bajo aguja)	0	288	0%
SST (Granate)	0	288	0%
CHL (Taganga)	215	73	74.6%
CHL (Arriba aguja)	215	73	74.6%
CHL (Bajo aguja)	215	73	74.6%
CHL (Granate)	215	73	74.6%
CHL (Cabo de la vela)	215	73	74.6%
Upwelling index (Arriba aguja)	149	139	51.7%
Upwelling index (Cabo de la Vela)	149	139	51.7%
MEI	0	288	0%
NAO	0	288	0%
Precipitation	0	288	0%
Total CPUE	166	122	57.6%
*Caranx crysos*	172	116	59.7%
*Euthynnus alletteratus*	176	112	61.1%

SST: Sea Surface Temperature; CHL: Chlorophyll; CPUE: Catch Per Unit of Effort; NAO: North Atlantic Oscillation; MEI: Multivariate ENSO Index; ENSO: El Niño/Southern Oscillation.

**Fig. 1:**
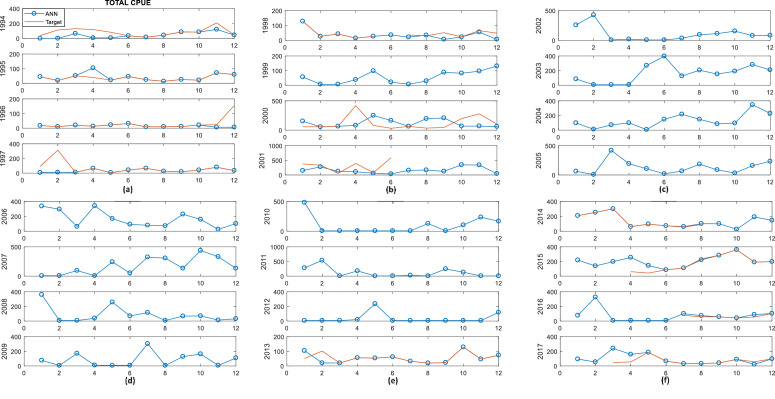
Time series of Total CPUE in red and CPUE with ANN in blue vs Months: (a) 1994, 1995, 1996, 1997; (b) 1998, 1999, 2000, 2001; (c) 2002, 2003, 2004, 2005; (d) 2006, 2007, 2008, 2009; (e) 2010, 2011, 2012, 2013; (f) 2014, 2015, 2016, 2017.

**Fig. 2:**
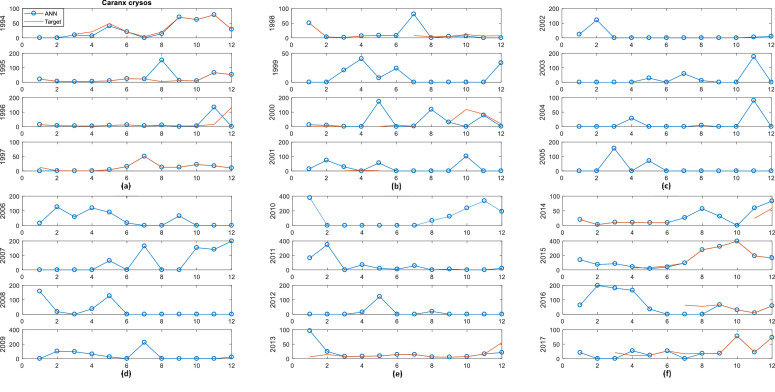
*Caranx crysos* in red and *Caranx crysos* with ANN in blue vs Months: (a) 1994, 1995, 1996, 1997; (b) 1998, 1999, 2000, 2001; (c) 2002, 2003, 2004, 2005; (d) 2006, 2007, 2008, 2009; (e) 2010, 2011, 2012, 2013; (f) 2014, 2015, 2016, 2017.

**Fig. 3:**
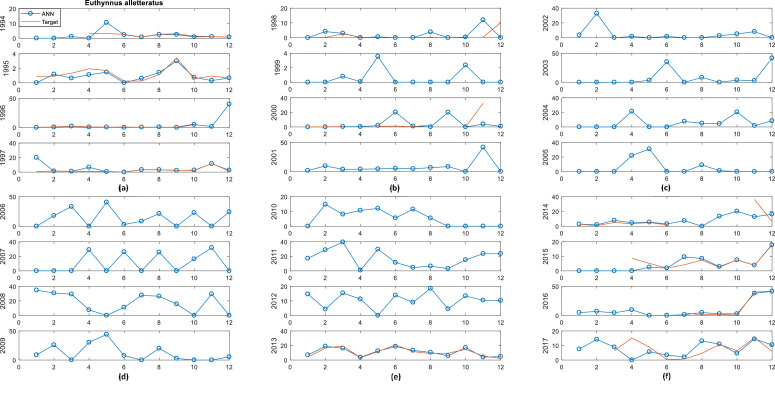
*Euthynnus alletteratus* in red and *Euthynnus alletteratus* with ANN in blue vs Months: (a) 1994, 1995, 1996, 1997; (b) 1998, 1999,2000, 2001; (c) 2002, 2003, 2004, 2005; (d) 2006, 2007, 2008, 2009; (e) 2010, 2011, 2012, 2013; (f) 2014, 2015, 2016, 2017.

The monthly time series from 1994 to 2017 of the historical values and of the reconstructed values for the fishery variables (Total CPUE, *Caranx crysos* and *Euthynnus alletteratus*), are presented in Figs. 1, Fig. 2, Fig. 3. The blue line shows the values reconstructed and the red line shows historical values. The absence of the red line indicates that there is no data in that period.

Table 2 of the supplementary material shows the data used for the training set of the neural network. This table shows the data of the network input variables (Year, MEI, NAO and Precipitation), and the data for the target (data available for the Total CPUE, *Caranx crysos* and *Euthynnus alletteratus* variables). In the supplementary material, there are 3 pdf files that contain the codes implemented in MatLab for the reconstruction of these fishery variables.

**Table 2:** Historical database of the beach seine landings correspondent to the species Blue runner (*Caranx crysos*) and Little tunny (*Euthynnus alletteratus*) in the period 1994 – 2017.

Source	Sampling period		Number of records /species	
	Year	Month	*Caranx crysos*	*Euthynnus alletteratus*
**PICEP**	1994	11	394	117
	1995	12	584	144
	1996	11	413	130
	1997	12	230	131
	1998	11	221	50
**Pargos**	2000	10	22	19
	2001	3	8	6
**SEPEC**	2013	12	337	426
	2014	8	272	150
	2015	9	976	205
	2016	6	395	183
	2017	10	793	359

SEPEC: “*Sistema de información del servicio estadístico pesquero colombiano*”; PICEP: “*Sistema de procesamiento de información de capturas y esfuerzo pesquero*”.

Table 3 of the supplementary material presents the original data and the reconstructed data with the neural network for these variables: Total CPUE, *Caranx crysos* and *Euthynnus alletteratus*).

**Table 3:** Sources and basic characteristics of the abiotic databases used.

Parameter	Period	Resolution		Source	Format
		Spatial	Temporal		
Chlorophyll-a	2011 – 2017	1 km	Daily	http://oceancolor.gsfc.nasa.gov	NetCDF
Sea Surface Temperature (SST)	1994 – 2017	1 km	Daily	http://oceancolor.gsfc.nasa.gov	NetCDF
Magnitude and direction of the wind (U y V)	2006 – 2017	25 km	Daily	http://apdrc.soest.hawaii.edu	NetCDF
North Atlantic Oscillation (NAO)	1994 – 2017	Punctual	Monthly	https://www.esrl.noaa.gov	txt
Multivariate ENSO Index (MEI)	1994 – 2017	Punctual	Monthly	https://www.esrl.noaa.gov	txt
Precipitation	1994 – 2017	Punctual	Monthly	http://www.ideam.gov.co/	txt

Finally, all historical data and reconstructed data are presented in Table 4 of the supplementary material. In this Table, the data highlighted in yellow were those reconstructed with the neural network.

**Table 4:** Correlation coefficient (R) for fishery variables.

Variable	R
Total CPUE	0.8590
*Caranx crysos*	0.9527
*Euthynnus alletteratus*	0.9529

## 2 Experimental Design, Materials and Methods

### 2.1 Study Area

**Fig. 4:**
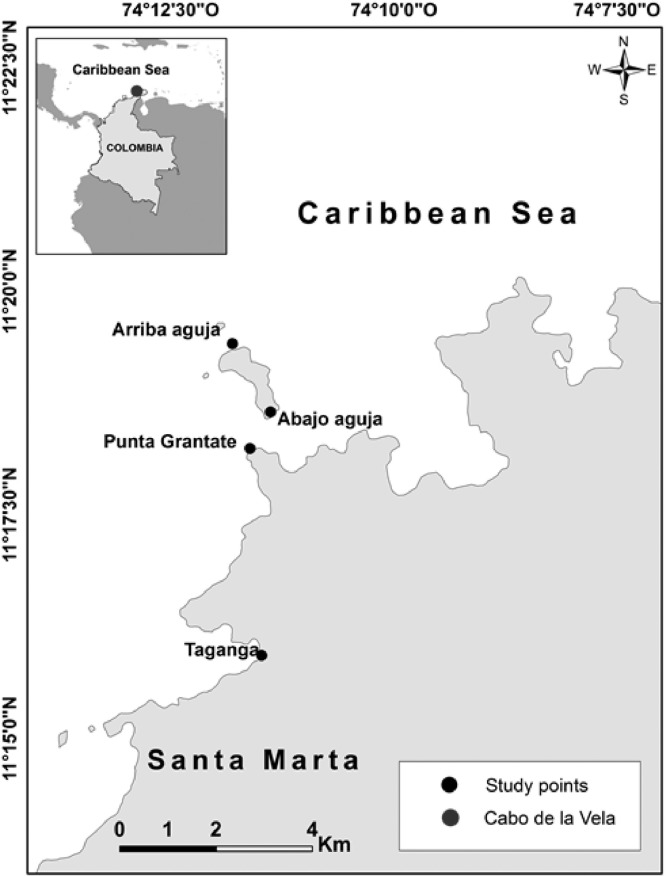
Map with the fishing area and the points where satellite information was acquired.

The coastal marine zone of the Magdalena Province between Taganga (11°16′03" N, 74°11′27" W) and Bahía Concha (11°17′50.5" N, 74°08′59.8" W), located north of Santa Marta, Colombian Caribbean (See Fig. 4). It is characterized by the exposure of the northeast trade winds and high biological productivity due to the nutrient load products of this phenomenon [1,2]. This area is influenced by two main climatic periods, the major dry season (December-April) and a minor dry season (July-August), which is also known as “Veranillo de San Juan” [3].

In the aforementioned study area, a great variety of fishing gears were used, among which the beach seine is highlighted. This type of gear is operated mainly in eleven “ancones” (small bays), located from the southwest to Northwest of Taganga (See Fig. 4).

Two historical databases belonging to the Fisheries Authority of Colombia (PICEP and SEPEC) that are hosted at http://sepec.aunap.gov.co/ were used to analyze the fishing landings. In addition, project “Pargos” information was used. The records of the statistical fishing information were carried out considering the methodology described by SEPEC [4]. These databases contain information from multispecies catch-per-unit-effort and for the species Blue runner (*Caranx crysos*) and Little tunny (*Euthynnus alletteratus*), which were obtained using an interrupted time series that includes the period from 1994 to 2017 (See Table 2).

### 2.2 Satellite data

The acquisition of data for the processing of abiotic variables was divided into three parts: oceanographic, atmospheric and indices. For the oceanographic variables, the daily data of Chlorophyll-a and Superficial Sea Temperature (SST) with a spatial resolution of 1 km were used, which come from diurnal measurements of the MODIS sensor mounted on board the Aqua satellite.

The atmospheric data were obtained with a spatial resolution of 25 km, from the daily average of surface zonal winds (U) and surface meridional winds (V), with the ASCAT sensor L2b 12.5 (The Advanced Scatterometer) on board the EUMETSAT satellite [1,5]. These data were reprocessed at a spatial resolution of 1 km. In addition, monthly precipitation data were requested from the Institute of Hydrology, Meteorology and Environmental Studies (IDEAM), taken by the station 15015050 located at the airport Simón Bolívar of Santa Marta (See Table 3).

To determine the characteristics and prevailing climatic indices for the area and period under study, the monthly data of the North Atlantic Oscillation index (NAO) and Multivariate ENSO index (MEI) of the National Oceanic and Atmospheric Administration (NOAA) were acquired (See Table 3).

To calculate the upwelling index, in the first instance the wind along the coast was obtained, by means of the geometric projection of the wind vector composed of its components U and V, which were projected in the direction of an angle determined by the slope of the coastline. Subsequently, the Coriolis parameter (Ekman) was used to calculate the transport perpendicular to the coast (upwelling index) of the “Arriba Aguja” and “Cabo de la Vela” points (See Fig. 4).

## 3 Data Reconstruction

**Fig. 5:**
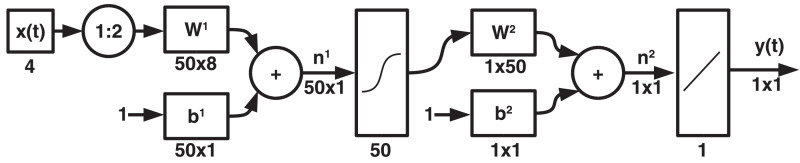
Dynamic artificial neural network.

To perform the reconstruction of the fishery variables, a dynamic artificial neural network was implemented [6,7] (See Fig. 5). A training set was formed with the variables Year, MEI, NAO and Precipitation as input data, and as a target the non-missing data of the CPUE total, *Caranx crysos* and *Euthynnus alletteratus* were used. To perform the training, only 107 data were available, which can be consulted in Table 2 of the supplementary material.

The ANN has two delays in the input layer, fifty neurons in the hidden layer, each with a hyperbolic tangent sigmoid (tanh) activation function and an output layer with a single neuron and linear activation function. To generate the estimates of the time series of the fishery variables, a network was implemented for each variable. Once the training was performed, the correlation coefficient Pearson's (See Eq. 1) was used to examine the approximation between the observed and predicted values (See Table 4).

(1) $$R\left(A,B\right)=\frac{cov\left(A,B\right)}{\sigma_{A}\sigma_{B}}$$

Where:

R: Correlation coefficient.
A: Variable.
B: Variable.
*σ_A_*: Standard deviation of A.
*σ_B_*: Standard deviation of B.

A value of R close to 1 indicates an adequate approximation by the ANN with respect to the target. The ANN data can be consulted in Table 3 of the supplementary material.

## Appendix Supplementary material

Image, application 1

Image, application 2

Image, application 3

Image, application 4

Image, application 5

Image, application 6
