# Supplementary material for: Reconstructed data of landings for the artisanal beach seine fishery in the marine-coastal area of Taganga, Colombian Caribbean Sea
Source: Data Brief. 2020 Apr 22;30:105604. doi: 10.1016/j.dib.2020.105604 (PMC7200242; doi:10.1016/j.dib.2020.105604)
Supplement: Supplementary file 2 [file mmc2.pdf]

```
function [Y,Xf,Af] = ANN_CPUE_TOTAL(X,Xi,~)
%
% Generated by Neural Network Toolbox function genFunction.
%

% ===== NEURAL NETWORK CONSTANTS =====

% Input 1
x1_step1.xoffset = [1994;-1.71;-4.7;0];
x1_step1.gain =
[0.0869565217391304;0.404040404040404;0.200803212851406;0.023809523809523
8];
x1_step1.ymin = -1;

% Layer 1
b1 =
[2.6228407571359282;1.8534233393064174;2.1130059295334624;2.5184906611837
481;-1.9554440799820103;1.7036461992826293;1.7987682867761812;-
1.4123387636286056;1.9307105406864484;-
2.1358840894694522;2.2160589878744927;-
1.6942732975838932;1.253635698184093;-1.1063100131625738;-
0.54160419475996335;0.045825127062251246;0.77401095747657278;0.0323873318
23817443;1.5861444617611391;-
1.5943274827452791;0.39169924399381534;0.45482511442656548;0.084262406550
565966;-0.48830990235820837;0.71055138575575938;-
0.16067362440332908;0.17705320698274149;-0.12517801310924309;-
0.63712049017663097;1.2220401371848368;-
0.086756999725737291;0.094190332365202759;-0.22407167689262214;-
0.22891200140575091;0.25976807739628777;-
0.70095670202834381;1.326047760068271;-
1.0097715418456814;2.2319014781802569;-0.91968591496709151;-
1.3209322872666514;2.5824412508943149;-1.8242859397766655;-
0.8152106310901639;2.0568527441252682;2.4384632564418687;-
1.5999022925771766;2.7803001468841027;-
1.7802782971117868;2.357560362675001];
IW1_1 = [-0.77764295191604327 -0.74430105034733296 1.1898143862886708
0.1750749320443909 -0.65399961403873941 0.17280026443262358
1.4179159706757334 0.31514668127467921;-1.6070164020665934 -
0.26808687584757046 0.26138343514868023 1.3107072421212342
0.41786207752358434 -0.99871231365999535 -0.19004735675608322 -
0.90824422856447884;-0.88288803099188373 0.024255788048732355
0.70105678598538768 -1.2319602153183142 1.2651696989200742
0.64969354468362528 -0.35781401207309993 -0.42133550580015849;-
0.20011805460188836 0.1549584723499231 -0.13038146218813684
1.4439119843272483 0.33070911164701983 -0.16655443418605925 -
0.5916390654928676 -0.6620720904275893;-0.50207369962903847
1.3311307453417203 -0.058142450826405609 -0.81724957775384077
0.37949154640562599 0.48807068470223797 -1.8355790594051939 -
1.043325010157454;-1.2072065090641166 0.86254474636271539
0.71644439142209915 0.016970841069335594 -1.2800141830133029 -
1.0495194875057792 -0.094828795073753122 -
1.4159429309780587;0.43415240517706438 1.1799718571115074
0.28866295371649914 0.91572542306627636 0.052315705168478131
1.2586150826254612 -1.0135712891114812 -0.57410825833740708;-
1.028335050763274 0.53026690869272042 -0.67031970282814179 -
0.90284036736878637 -2.5084365574622907 -0.09553480308432992 -
1.2645550577981408 1.6516098430879602;0.1738330992137769
```

0.29446989227240405 -1.0490808375446208 -0.41479062233406866
1.2643820650824189 1.1241207685273371 0.98047832089595144
0.33067892627569628;0.88525091213870377 -0.35427732814054308
0.60597726649716355 1.3101871287466429 0.8112842964900856 -
0.63351707290354387 -0.05829338116289396
0.44021032684499511;0.026742646323875578 0.019888535326248416 -
1.1300123802451745 1.2064869431536722 -0.53581494211146119 -
0.71632304942897995 -0.49410674570493818
0.07749066483234357;0.97829560336198962 -0.22789723921997446
2.0199580634569312 -0.88884203217930846 0.41735672990228229
0.37818624522139732 0.51257984917306654 -0.030871594726018227;-
2.2188139162342648 0.50118949807210422 -0.88552308737304597
1.729499386074649 -0.65039796818542017 0.9624566238896447
0.60849933123220434 1.1741381058657669;1.401295214963131
0.0065928903477725119 1.101139962484343 -0.73207815297980805 -
0.74839943444053469 -0.22767844879849661 0.45550258157526635 -
0.092201796697630997;0.29265106281183773 -0.48460381021376181 -
0.016981983827190811 -0.39579765197962052 -0.89676243061446947
1.0004716921643462 -1.3667086762670795 0.99436506350087728;-
0.75102502811726501 -0.4335566334031471 0.5749950787856668
0.58094150104166131 1.9171278882428535 2.3750630635969769
1.6992539438515424 -0.64821667849670028;-1.5538086627156094 -
0.0085301492187629339 0.79227358462294306 0.19512031108677574
0.30644625106638307 0.057144987961185556 -2.0699027185828927
1.3437320808245423;-0.40328353947838591 0.058052158851464121
0.51108825218361253 2.0652486195224804 -0.71577302012799426
0.073648217748311137 0.17050450364440803 2.083867684616465;-
0.97351475497544815 1.4375924861534786 -0.21221597961571267
0.24653896040700685 1.9074095098754524 -0.49805674827378482 -
0.67678663648598403 0.4510976095879729;1.2483174504587653
0.4468023120144255 2.155461130695377 -0.31843883805393092
0.15222851419504993 -1.537936344701557 -1.8371120358194544
0.53168321001523422;-0.65919666878063143 1.0200094433176172
0.026496137119301259 -1.6243890090335154 0.37977066159542799 -
1.865804272337614 -0.25041547306195522 1.4160276970210306;-
1.4173071205069088 -0.64842794279806504 -0.5724032859558319
0.48985672510918798 -2.510818581557742 1.4371505987776561
1.1938905472331545 -1.3041982043189353;-0.58515907444883974
0.65878220759681583 -2.2739997804617742 0.55664776823063378 -
0.59045961452947038 -0.37043287168147043 -0.7644525961415799 -
0.15562745253231414;0.36443846448220168 0.37936753766101433 -
1.389890164675835 0.66883366837060898 0.041394538299582825
0.93706331769069928 -0.49308296596767559
1.2557888892359919;1.0196525145599191 0.355927419426811
0.08268179941583477 -1.2029670123571612 1.9270364158680262 -
1.1169062046914247 0.2707385857277948 0.71393504042164058;-
0.67398368413260856 0.47838480116258308 0.11720139278010849 -
1.1894165449997827 0.089208677257952423 1.5590442547934296
0.36709532915347215 -0.81103038753250367;1.2189124272492167 -
1.8080887893938919 -0.26319206426692343 -0.21421429572659395 -
0.034034838410900568 -1.6817769807945628 0.4134896666100123 -
0.1661158344491421;-1.4655310745067656 -1.040100333101716 -
0.30959499630520937 -0.7611743018997813 -0.17565628294575236
0.32859334080770963 -0.50630129023920079 -
0.81672472391906292;0.26806565769125151 0.22491647377813317 -
0.86796963556030693 1.840224197321225 -1.0729160173498153
1.2576316459142038 0.29015409344119442 -

0.11303034668380776;0.82027204483388449 -0.84793007128437614 -
0.98740147414248869 0.48190777791356804 -0.62927986964987526
1.6416498034594291 -1.5938042080667179 -
0.8697133008013197;0.71510752834110203 0.46225193228963374
0.1182261706958137 -1.1509254422356012 0.59518669684080239 -
0.60322914108049852 -1.7370591890567089 -
0.29244700817563724;1.1065812856926844 0.76582731711491692
1.4640478495599965 -1.5142205333649708 1.1449367222184308
1.4883136446662861 0.28421171886811603 -
0.31782858258208835;0.22556315687377182 0.57070431257067999 -
1.5334262022550174 -0.55873700952263916 -1.2640040129209069 -
0.16321335713111723 -1.1155159679272713 0.5857773093848706;-
1.0916847792612587 -1.429868689005106 0.66375468397054249 -
1.9495757198642008 1.1031683306773454 -1.4105248960016616 -
1.4762370871759611 -0.88355495322832389;1.9386125454177783
0.77859860998249064 -1.2225544077805646 -0.21306510136303755
0.52357658706572574 0.40232397016081151 -1.8371074446929365 -
1.0446873292371746;-2.207829035463325 0.9415542455368352 -
1.0316173152978536 -1.3896387361234581 -1.6139980516871986 -
2.8906561251450591 -0.8497396182507585 -
1.3439601816869402;1.5986306579829253 0.52263555334044676
0.22189212048283843 -0.75603467670159663 -0.8818139860366293 -
0.85315677362491416 0.10784485806864759 -0.27976859843329333;-
1.363855280945891 1.3869184696296701 0.085873025263100658
0.43515612608558046 0.98546834548944118 0.16615346685702975 -
0.94824221327417935 -0.21928608976640268;0.79371068220591023
0.27782403319021054 -0.52769210624118612 0.26491052775195995 -
0.24402338979249788 -0.93736798894580975 0.17238698438494993 -
0.98554632314000123;0.1292892960573773 -0.77520774058692066
0.80269304678294084 1.1002387290183335 0.72491261972164756 -
1.5255616280201187 1.7935291572573602
1.2995446839024467;0.014102671549615727 0.86306011881701683 -
0.0078789715376784281 -1.5551829380161435 1.2890114202291212
1.513012654169823 0.33792206317344248 -
0.10866281843157705;0.74370932472297546 0.10569233475312653
0.6305183117113119 1.0458829633261875 -1.8286923757447584
0.47798205832849716 -1.4675266780889058 0.57010003185619085;-
1.3605382948306479 -1.0630096573917118 1.1050761978378643
0.43283784070193854 0.12147258370166393 -0.59428056554834918 -
0.034788628583809697 1.4576784963763063;-2.3996236277418466
1.4785085130088611 0.79778138341567861 -0.53987177940811559 -
2.2772028845076 0.67078435830300942 0.85252705791448669 -
0.97676949383492329;0.68253304847041329 0.035331618935896897 -
1.0146612894686797 0.94170448729913292 -0.6336103017677801 -
1.0361178824310779 0.4567067749150614 -
0.58374491318015787;0.93088772566542588 0.6135891208101969
1.4173063515415647 0.81782826414042709 0.075880335947910119 -
0.16185858062238473 1.3212351676118528 -0.6560904136671295;-
1.3559465332419474 1.6840298622921523 -1.2844649503550187 -
0.51588357924644856 0.20229830926510931 0.25866760001672162
1.2467512375432794 -0.097267892233583453;0.41693998399046461
1.0603614918682516 0.029484187493403549 -0.77102407878399748 -
0.37461819518931239 -0.96766340247079041 0.20783080624831146
0.26408901535653834;0.067629410843018892 -0.84841752656309732
0.48949101629281522 -0.031739603735518589 -0.030148813360576596
2.1163093180555101 1.3024093416534073 -0.89160287132285287;-
0.053986913396112814 1.2399180223012007 -0.054236596675888198 -

```
0.40019432445717712 0.47636152353964051 -1.3542813672192224
0.2502136518893805 -1.0151055853944786];

% Layer 2
b2 = -0.60989437490383636;
LW2_1 = [-0.79192032568780568 0.87222757714803445 0.1849849466959847
0.29053256690764634 -0.59927529103871791 1.1354848480282667 -
0.40994426969237863 -1.368234250124349 -0.51188140154845574 -
0.41784099470960689 -1.437280492098052 -1.5170344194600531
0.60292653142302899 -0.33278225260039912 -0.49325409355080369
1.0100199874677451 1.51060230635198 1.0584434631178867 -
1.7029019555909379 1.3074474407961456 1.4974253248947393
0.8051885498868534 -0.094258705127357151 -0.26185133965811075
0.68673902551119204 0.68346359819093527 -0.87770091666823258
0.54435510676913834 -0.29115841450855601 1.2649818656264864 -
0.49121787698818947 -0.36820242555380206 0.57027339599531923 -
0.85341286063794741 1.3401245357815756 -1.8119275156484957 -
0.078393040232093245 0.48309536031258304 0.30170504912781354 -
0.92997357392450275 -0.82630276351614074 -1.9223294345044535
0.63649919026146762 -0.53148934134380499 0.079134475368999896
0.14590369251048271 0.36948450850598641 0.058482191859297303 -
0.018674512240889573 0.73963772350805901];

% Output 1
y1_step1.ymin = -1;
y1_step1.gain = 0.0056463839145815;
y1_step1.xoffset = 6.744;

% ===== SIMULATION ========

% Format Input Arguments
isCellX = iscell(X);
if ~isCellX
    X = {X};
end
if (nargin < 2), error('Initial input states Xi argument needed.'); end

% Dimensions
TS = size(X,2); % timesteps
if ~isempty(X)
    Q = size(X{1},2); % samples/series
elseif ~isempty(Xi)
    Q = size(Xi{1},2);
else
    Q = 0;
end

% Input 1 Delay States
Xd1 = cell(1,3);
for ts=1:2
    Xd1{ts} = mapminmax_apply(Xi{1,ts},x1_step1);
end

% Allocate Outputs
Y = cell(1,TS);

% Time loop
```

```
for ts=1:TS

    % Rotating delay state position
    xdts = mod(ts+1,3)+1;

    % Input 1
    Xd1{xdts} = mapminmax_apply(X{1,ts},x1_step1);

    % Layer 1
    tapdelay1 = cat(1,Xd1{mod(xdts-[1 2]-1,3)+1});
    a1 = tansig_apply(repmat(b1,1,Q) + IW1_1*tapdelay1);

    % Layer 2
    a2 = repmat(b2,1,Q) + LW2_1*a1;

    % Output 1
    Y{1,ts} = mapminmax_reverse(a2,y1_step1);
end

% Final Delay States
finalxts = TS+(1: 2);
xits = finalxts(finalxts<=2);
xts = finalxts(finalxts>2)-2;
Xf = [Xi(:,xits) X(:,xts)];
Af = cell(2,0);

% Format Output Arguments
if ~isCellX
    Y = cell2mat(Y);
end
end

% ===== MODULE FUNCTIONS ========

% Map Minimum and Maximum Input Processing Function
function y = mapminmax_apply(x,settings)
y = bsxfun(@minus,x,settings.xoffset);
y = bsxfun(@times,y,settings.gain);
y = bsxfun(@plus,y,settings.ymin);
end

% Sigmoid Symmetric Transfer Function
function a = tansig_apply(n,~)
a = 2 ./ (1 + exp(-2*n)) - 1;
end

% Map Minimum and Maximum Output Reverse-Processing Function
function x = mapminmax_reverse(y,settings)
x = bsxfun(@minus,y,settings.ymin);
x = bsxfun(@rdivide,x,settings.gain);
x = bsxfun(@plus,x,settings.xoffset);
end
```
