# Supplementary material for: Reconstructed data of landings for the artisanal beach seine fishery in the marine-coastal area of Taganga, Colombian Caribbean Sea
Source: Data Brief. 2020 Apr 22;30:105604. doi: 10.1016/j.dib.2020.105604 (PMC7200242; doi:10.1016/j.dib.2020.105604)
Supplement: Supplementary file 3 [file mmc3.pdf]

```
function [Y,Xf,Af] = ANN_Caranx_crysos(X,Xi,~)
%
% Generated by Neural Network Toolbox function genFunction.
%

% ===== NEURAL NETWORK CONSTANTS =====

% Input 1
x1_step1.xoffset = [1994;-1.71;-4.7;0];
x1_step1.gain =
[0.0869565217391304;0.404040404040404;0.200803212851406;0.023809523809523
8];
x1_step1.ymin = -1;

% Layer 1
b1 = [2.2044458942594303;-2.7401582935733617;-
2.5689899816807098;2.0385331579306523;2.6877432421263716;-
0.9250224000575592;2.3067767344397812;-
1.7808387037242204;1.2721063820527054;-
1.3165253455684034;1.483138186486906;1.5343689298399164;1.483942138471753
7;1.4078261006645476;-
0.89071413316597359;0.10745221377150178;0.97249617813519618;-
0.82101652462302543;-
1.0487992198886695;0.3510698421128095;0.31921758363320185;0.0377887162830
16048;0.13310456565367662;-0.55418268424034189;-0.051341704254453335;-
0.48350508491241195;0.4209192268188417;-
0.29383772149904819;0.31178930323489967;-
0.20980576049692942;0.43462999226600851;0.82060255343454369;-
0.68450460449560735;1.0136880860116064;1.058066521597455;0.79948443008537
24;1.1711723151218023;0.76389623570277743;-1.2809289663813757;-
1.7750748511719299;-0.90300602412180997;-
1.765021608749139;1.7008767097489357;1.7374470782230806;-
1.9765955180490433;1.9116279535202665;-2.8532602564586771;-
1.9922971067900943;1.7355540652824417;2.3074069606528997];
IW1_1 = [-0.71004896456880473 -1.1851492881666419 -0.018361830593622428
0.38995007009686317 -0.52273197817362138 0.50148174342053486
0.75731576525971134 -1.5369249411378154;-0.46240540961600402
1.0026331867871279 -0.70890923486642277 0.090322080381073158
0.22817110441415592 0.82813439737448757 -0.48590665265983185
0.92816275080176414;1.101345637161077 1.1791281130809019
0.80162615029427675 -0.67712481036837169 0.40480048333487567 -
0.26235785277034934 -0.93215498743240033 -0.54237305346775067;-
0.74493940090740118 0.77345171351562769 -0.78679970863860482 -
0.72391450503022758 -0.7775783834375386 -0.53959997051272146
0.4647985013783093 -1.3446226700067958;-1.5288863117176685
2.6824509972952577 0.62150835769363888 -1.1337604766519458 -
0.032005388465941816 -0.25276704727059923 1.4651490448072955 -
0.25930792741663183;0.42714208711229357 -1.2320945126476133
0.71000227983449604 -1.039001516438897 -1.2782445874847108 -
1.5561791990817739 -0.37761643511807208 0.8549909720802944;-
1.1896557318071366 0.35138651378794161 0.010948461225258117
0.4356958316404213 1.2997226541385341 -0.13636662604743721 -
0.41748997418515077 -0.61971561924977903;0.13009013226562557
1.2682753247796072 0.79952678641499209 0.69391859907833509
0.43090951519150705 -0.30909105290455435 0.94849210407957008
1.4230711127641384;-0.64667680204676392 1.3800880238114641 -
0.09988995272217259 -0.69192954074537849 1.0951745103094048 -
```

0.20566444880989149 1.2425297529598498 -
1.021644650086277;2.0756292311669369 -0.78550669179370469 -
1.3814390388829578 -0.3047569407876119 -0.30335418912842871 -
0.24473470311644865 0.84337352554435152 0.11586907996348378;-
1.1346857530380925 0.38013090154129969 0.46573619622646989
1.1736386995112975 0.077404563309236979 0.5983213086337692
1.7516879073700353 1.0113692307746811;-0.91445722141066144
1.4764367052168827 0.3711136872924487 0.61738282436160852
0.49847292250616548 -0.88304307275133176 0.3836902895222723
0.5298872269361582;-0.38359941945280335 -1.9199145344653332
0.70932494158754555 -0.29836343857567771 -0.38103647087683978
0.14689189174781167 0.59444851746910343
0.68689911521159353;0.2358806570465346 1.8524546663010404
0.050986074600035534 -0.5350685799484437 -0.1342824120761002 -
0.34788983952862146 -0.099742625088073036
0.91951748486258467;0.92858342199839594 -0.85795427297420734
0.50537076981577445 -0.95714116977303865 -1.3118912884981553 -
0.89155079822111427 0.1948383658607441
1.0935158485537622;0.53266329181618888 1.4087772188148031 -
0.65703217446546425 0.027565153832254993 -1.1381447122181907 -
0.48861055341109111 1.1690235144812162 0.21444615251507743;-
0.5238767624005668 0.29001220261683547 0.63111722830713024 -
0.023411776656421999 1.3931989611678821 -0.9675777473820163
0.42110448281295887 -0.13516954925884922;0.49968293642638262
0.69325421630303563 -0.17448077761370195 1.4038260733458903
1.3513359113914791 0.85880374066563703 0.46738204723128679 -
0.3411359919886327;1.1437866932630265 -0.4877741378424344
0.32957939554899002 1.0553703352940276 0.34863892463084911
1.0762359260582819 -0.99669891507083508 0.84925363880148697;-
1.0172370848630752 -2.2144661840378874 0.20505513281974824 -
0.61828580957869717 -0.170426866039009 -0.31049209922505389
0.7505557880892566 -1.1003655747527372;-0.84744918680494952
1.0834582310924545 -0.39062948020820465 -0.013441357001146676
0.26412528232451676 -0.25177437749967857 1.0024729728822599 -
1.295940016612988;-1.6820540718812713 -0.26658550456352692
1.1817280393102376 0.20091555197720487 -0.94865236771247108
1.0085806127859815 -0.97269017950936565 -
0.58048947450117205;1.2215434703056032 -1.5458742705903392
0.61657648489419536 0.26913445191010527 0.13594133018535437 -
0.34121620153642396 0.32834610908245904 0.97907274504066422;-
0.26908786922551814 -0.3900644874057333 -1.2839929918392634
1.2028086322592682 1.2959740041901275 0.6036382836826274 -
0.57352738006181458 0.81290124463068725;-0.59546126707125369
0.39336045233363065 -0.83942658284038807 1.7182136984126948
1.1286647414797641 -0.65162614078334202 1.3942536429785162 -
0.61601999035796207;1.1346583073569985 0.86887912498015163 -
0.018453025892257918 -0.65881910241235886 0.67674945483215554
0.058845444217307794 1.1046511424667436 0.04770625948290598;-
1.1852204123292895 -1.1788712840819524 -0.11178206786021734 -
0.94725740878453657 0.11976613910419433 -0.46842080355961324 -
0.66275391912653348 -1.8617735366737376;-0.053007208597721511 -
0.41850470763305397 1.2430330034757426 -0.31901254754787733
0.48220114562230887 0.25446671731537318 1.6102052681679495
0.34473344559753788;0.35997766139749937 -1.3625129382581782
1.7789943712351428 0.35834211098832885 -0.66147500473138576
1.2219891920480159 -1.2520984639570465
0.10017870740638413;0.020736511710055031 -0.055239452501162793 -

```
1.555679586192463 0.59723295662464615 -0.028210248111543389
0.96244994242916626 2.9004430383312334 -
0.18305509905490103;0.63669741309704364 0.043489656520372809 -
0.15271299536969365 -1.6046790217778917 -1.1208553479303196 -
0.078051642140783584 1.0973731410862435
0.59825835843735142;0.69718067080272095 -1.5409886597658038
2.0466411851712083 -0.19494165289023788 0.9312439502414146 -
1.120426091399285 1.241234240153219 -0.19222975490966498;-
1.239530568482881 -0.65751794392929397 -1.172442882013939
0.54817646929779584 0.11494559158908671 2.0918737955545481 -
0.94254969544174572 -0.6374819208471233;-0.16547929508287149 -
0.89570240360032038 0.019066190158688139 -0.68312709586586118 -
1.0175511296372834 1.1099067044606274 1.1227997786959938
0.26837971850551923;0.85302942660696468 -0.37032775930251505
1.2521911855700538 -1.1760065948682994 -0.27587930489845319 -
0.44192110386321298 1.1057087824720637 1.3490263122470285;-
0.24626485179650257 -0.73351781880671585 -0.043652657801884605
0.65062924432707581 -2.0999988035500001 -1.3531361441768663
0.22928078949024144 -1.0229461155596882;0.42268403886442757 -
1.2026305725849771 0.38319689009253621 0.417486489145941
1.2430792969414826 0.81847774670288609 -0.53729566823208996
1.243675939996989;0.73626114574089052 0.9441527478905537
1.4944419062741519 -0.017226312541332812 1.0882940827076588 -
1.0177598618141153 -1.0821607878668453 -0.11000529668985327;-
1.28271377850866 -0.16820182819706664 1.2393931800970153 -
1.7192947496510624 0.35996226211667531 -0.95633501564012646 -
0.5018266383271931 -0.073487270937610524;0.010792441369905554
0.11553005796143379 -0.62664399350033861 -0.65682796709102953
0.025402875626726027 0.090418901856363643 1.3915262434175766
1.0328294633381316;-0.86964218977778762 -1.2686117547106464
0.45565048978787914 -0.7487368468199791 0.63710484278381307
1.0135795527852023 -0.40695608423654311 -0.58865138539131689;-
1.9447960592381071 0.010869830890996609 -0.42250281660902594
1.5903333402948432 -0.49889221700917974 1.4904544852672115 -
0.12518659074486937 -0.70672387572793649;0.52625974970764056 -
1.0029750285881351 0.63341558587876556 -0.50039184286822047 -
0.929701140932223 0.18043773811068373 1.0402342508505444 -
1.0768885964871482;0.9705575661626562 0.53908241657223854 -
1.2252626235382691 0.14091332787649175 0.74807520445224307 -
0.76771270213541476 -0.63713662142464367 -0.86278391695975876;-
1.0305016481133993 -0.10371020481823831 0.20149791252132182
1.2438534849526981 -0.20840925758977891 0.48971673306388308
0.92658352184093973 1.1850854854049835;0.89153967692452318
1.3267801660046414 -1.1987059211699684 -0.35381514655768209 -
0.049963759629897679 -0.41635605017660382 0.16981475149309888
1.1677095337763879;-0.29804136820708721 0.51203534825522501
1.45654161738543 -1.5493458035966525e-06 -0.98037897185353307
1.4416215342722805 -1.0788014834000987 -1.3700816804606755;-
0.15805499883924332 1.6328791425252893 0.96912643231946982 -
0.97423632472763988 0.9301134583557279 0.4066831055558211
1.0571338017788769 -0.17072635396790051;1.3039940084625903 -
0.33800570174885736 1.150504749813847 -0.34372792394739599
0.15658833148164275 -0.40626640803927744 -2.033949979286489
1.1873085745617757;1.2232761023434062 -0.60844298256866292
0.61746932701408219 0.75272926084706648 -0.99806660235656175
0.68955377923982819 -0.85136708939901629 -0.33450993340120921];
```

```
% Layer 2
b2 = 0.035048438622540527;
LW2_1 = [-0.40177398575811551 0.096714758642746257 -0.58693603106359549
0.94375651268293137 -1.3885134097833309 -0.22025085472527919
0.38005547820170504 0.47591701910148992 0.77200697778936567
0.77164606764741461 -0.76603821871053446 0.6853465512669854 -
0.32258503100015845 0.72334102331381556 0.13962267184203722 -
1.7089935907390217 0.061556342934565363 0.039637693669989885
0.72213271457641282 -1.065000813313169 0.065258380231713944 -
0.65884118773155376 -0.44397894587644204 -0.29357017117478912
0.79773145261604383 -0.44237611776352065 -0.74274413793542848 -
0.61941039261809072 1.0617424826430502 0.50958133844400022
0.38363295137475584 -1.2772838827109472 -1.4600016217981209
0.54545447467042374 0.73371853372549412 -0.038466761633668946 -
0.55737061709018709 -0.57086159927910318 0.58517129507694443
0.34217681463019672 0.24648136991904668 1.0592145515816198
0.19832330473733589 -0.65337061455121048 -0.56258364422499785
0.32140862316007435 -1.1324872129684695 0.47237469527533166 -
0.75068973079289503 0.20651837576260512];

% Output 1
y1_step1.ymin = -1;
y1_step1.gain = 0.0050467836847577;
y1_step1.xoffset = 0.043;

% ===== SIMULATION ========

% Format Input Arguments
isCellX = iscell(X);
if ~isCellX
    X = {X};
end
if (nargin < 2), error('Initial input states Xi argument needed.'); end

% Dimensions
TS = size(X,2); % timesteps
if ~isempty(X)
    Q = size(X{1},2); % samples/series
elseif ~isempty(Xi)
    Q = size(Xi{1},2);
else
    Q = 0;
end

% Input 1 Delay States
Xd1 = cell(1,3);
for ts=1:2
    Xd1{ts} = mapminmax_apply(Xi{1,ts},x1_step1);
end

% Allocate Outputs
Y = cell(1,TS);

% Time loop
for ts=1:TS

    % Rotating delay state position
```

```
    xdts = mod(ts+1,3)+1;

    % Input 1
    Xd1{xdts} = mapminmax_apply(X{1,ts},x1_step1);

    % Layer 1
    tapdelay1 = cat(1,Xd1{mod(xdts-[1 2]-1,3)+1});
    a1 = tansig_apply(repmat(b1,1,Q) + IW1_1*tapdelay1);

    % Layer 2
    a2 = repmat(b2,1,Q) + LW2_1*a1;

    % Output 1
    Y{1,ts} = mapminmax_reverse(a2,y1_step1);
end

% Final Delay States
finalxts = TS+(1: 2);
xits = finalxts(finalxts<=2);
xts = finalxts(finalxts>2)-2;
Xf = [Xi(:,xits) X(:,xts)];
Af = cell(2,0);

% Format Output Arguments
if ~isCellX
    Y = cell2mat(Y);
end
end

% ===== MODULE FUNCTIONS ========

% Map Minimum and Maximum Input Processing Function
function y = mapminmax_apply(x,settings)
y = bsxfun(@minus,x,settings.xoffset);
y = bsxfun(@times,y,settings.gain);
y = bsxfun(@plus,y,settings.ymin);
end

% Sigmoid Symmetric Transfer Function
function a = tansig_apply(n,~)
a = 2 ./ (1 + exp(-2*n)) - 1;
end

% Map Minimum and Maximum Output Reverse-Processing Function
function x = mapminmax_reverse(y,settings)
x = bsxfun(@minus,y,settings.ymin);
x = bsxfun(@rdivide,x,settings.gain);
x = bsxfun(@plus,x,settings.xoffset);
end
```
