# Supplementary material for: Reconstructed data of landings for the artisanal beach seine fishery in the marine-coastal area of Taganga, Colombian Caribbean Sea
Source: Data Brief. 2020 Apr 22;30:105604. doi: 10.1016/j.dib.2020.105604 (PMC7200242; doi:10.1016/j.dib.2020.105604)
Supplement: Supplementary file 4 [file mmc4.pdf]

```
function [Y,Xf,Af] = ANN_Euthynnus_alletteratus(X,Xi,~)
%
% Generated by Neural Network Toolbox function genFunction.
%

% ===== NEURAL NETWORK CONSTANTS =====

% Input 1
x1_step1.xoffset = [1994;-1.71;-4.7;0];
x1_step1.gain =
[0.0869565217391304;0.404040404040404;0.200803212851406;0.023809523809523
8];
x1_step1.ymin = -1;

% Layer 1
b1 = [2.6911266840034416;2.1548872871653915;-
2.3886068033941386;2.1393478547089697;-
2.1737934277985551;1.7056689617631338;-
2.5161479310284194;1.5326636651943175;2.2466602593210054;-
2.0167326389016536;-0.927109867177767;1.7843174432324518;-
1.4285188055558136;-0.81587966141813595;-
0.93723293938887342;1.0931372619370945;0.85122749767998418;-
1.1063758933469949;-
2.0880771549179524;0.32929954902740899;0.61676134924823289;-
0.2619412226200028;-0.035571829503786845;-
0.16560024633708728;0.95245606013114048;-
0.2526360768725826;0.82886533674004725;0.65705608170412444;-
1.0505586610025588;0.31079820735755981;0.49323212896430252;0.807365075024
6032;-1.229590048377645;-1.0727895551883451;-0.42227936825806489;-
1.5233083163125651;-1.4284639981836722;1.6898574149701111;-
0.82465177660234656;-1.6363954277148238;-
2.240599302231129;1.9835945724236739;2.0355077466914628;-
2.7591780691825356;2.5645087246185891;-1.1595600945738123;-
2.0027969614418755;2.1630395203289075;1.5120443236663803;2.23934139917707
28];
IW1_1 = [0.32409374556730242 -0.033213950333891094 -1.2067171244484223 -
0.17638488833368954 -0.93854948782464875 0.42364430402103342
0.79786220409772535 -1.1171247070335648;-2.2302357589717112 -
1.4296547471587457 -0.22056085203473741 0.1605443554071348
0.72050177179436159 0.8946239254284164 0.72495337781144198
0.20698319112184135;1.0379950376511049 -0.23323211440811642
1.1335346034831066 -0.9758954282787744 -1.0964373648797545 -
0.078049496261365953 -0.77195949883709414 0.34513497416578792;-
0.62481417632984571 -1.2383065848794486 -0.58278416962749735 -
0.8969470651967405 -0.21347758826904831 -0.64693808428269961
0.10307487721808739 -1.0380829909372218;-1.2143254543418167
1.2772132540244343 0.42308862992556945 -0.67694785052408191 -
0.22820599312644005 0.1609324868311349 -1.8316895374028572 -
1.2887525988562727;-1.4151559776397591 0.2878353853151423 -
1.4841710383752831 0.23301728278869713 1.0667027816223766 -
0.29632207213263723 -1.0714061380448117 -
0.0071257436332652O1;0.019682329143232456 -0.56590139948802165 -
1.0643015620109442 0.33226399437673104 0.55873757991506212 -
0.73069164652690011 0.62629405134618332 1.3541217368486025;-
0.48721187398656329 -0.26608605452244999 1.1117544315675694 -
1.1181215448191182 1.2198065569923036 0.47130774824831945
0.65066913526138603 -0.71472964874259415;-0.13056178078178962
```

0.82793239416154496 -0.11545499411709248 0.12296278576967538
1.9588780265977306 -1.0551555566845994 0.26236139454775692 -
0.19996007298140855;0.97237026250066638 -0.63065228603768353
0.6159852090604151 0.029670025461215006 0.32050684852964922
0.76726761553551137 -1.461569213952695 2.1287982127676499;-
0.24391938841571217 -0.74554226725073236 -1.2067074358613312
1.1911291645405924 -1.6301547771690097 1.8166689085147916
1.6476792715383981 -0.86071848587654221;-0.44784884814998821
1.2191533719085874 -0.17114705129273231 1.4171493327885494
0.89828162431763259 -1.4866197481900394 -0.53630251695378772 -
0.63803325362961938;0.27931536895400233 -0.37422876288513801 -
0.62328988022117504 -0.15209506856321167 -1.5113733423034124 -
0.3429361201382794 0.38915213180072716 -1.2427887729395171;-
0.62030476191347206 0.20274790241223001 -0.43478954714924278 -
2.514115255237388 -0.43221849338419088 -0.80471575117195016 -
1.7642993452224078 -0.98369510286136419;0.91019292555125697 -
1.3058747886501274 -1.337302686525343 -1.6283287112131051 -
0.12906345394742258 0.32949964102490492 -1.0158325521961289
0.1087266889615958;1.2957881670102653 -1.3736131032309284 -
0.60736207272818366 1.1152525411510821 1.9133244593695766
1.5793257450637073 -0.088550003458189724 0.58605361317955518;-
1.0525483540497225 0.75660842237454595 -1.6750357649727698 -
0.2390935326116434 0.30063892164773348 -1.1790942560747202 -
1.6759740230621796 0.23151373357414765;0.54941562605688077
0.28287873383777123 -0.40883037570062986 -1.0622538438542592
1.4533212674606015 -1.058551523867538 0.39517221221123083 -
0.53726693267605086;0.68738114963959385 0.76464514167489706 -
0.087089567450443384 -0.24933475484230769 0.20192908146390867 -
0.77413102916621157 0.96432204442077174
1.0261438006992147;1.8581294033486959 0.46776089748538863 -
1.0934682927669781 0.79507565768978639 1.3955737553961021 -
0.46837934899402156 0.1126560994026056
2.6181257817929193;0.39789766386511988 0.2301149356522442
0.79423936323017641 -0.44933618873490844 1.6716573689754428 -
1.3569420942161952 1.0396792380364714 -0.41618950279961098;-
0.8450065645264907 1.2755112853262112 1.0230532564408381 -
0.87936427500500647 -0.10335957375367302 2.353210613705234 -
0.096675087828551359 0.85369942727791348;1.0576399989799741
0.69160864414070256 -0.82838190787080868 -0.44504495203535743
1.2903283059235899 -0.77708222960247408 1.0626217672425651 -
0.87695220192575685;0.53831804894655566 0.77483112000964793
0.8576268796796066 1.0238433233095159 -0.8197563917374755
0.26933359610526952 1.5866613062446797
1.2258330114369915;0.44937217750555392 2.0786682164466934 -
1.2794931831411349 1.2844591578186777 -0.75137174464753576
0.060849502164312015 -0.85202772265603666 0.27457473237979702;-
0.91891470036785972 0.63516039483727926 -0.36777925576290099
0.77012771755775511 -0.83156084592767698 -0.37888689627565814 -
1.0124078823725307 0.063015357050966878;-1.1630067886642192 -
1.1377126199436907 -3.1239490285156331 0.89715802069024464 -
0.82711827034771901 -0.94583168977141807 2.239152327173076 -
2.1566055846834322;0.50851271003811505 0.81969925505813679 -
0.22212003996951635 0.44618376583390418 -0.72596940679724409 -
0.52094680737155541 0.39605868345211515 -
0.22135593873658796;1.7686555693853379 1.5118694441255685 -
0.0072690685277377043 -0.27339658045868076 1.1277990086721525 -
0.59834840372710396 -0.4329247091018969

2.4864329121952817;1.5121131491934752 -0.69854878193047176
1.9092460836461751 1.3345976063901275 2.8931355966754713
0.26444268694239642 -2.283893858063875 -
0.14738912281997846;1.2317308459086245 -0.88694891535002807 -
0.073540550301072524 -0.57435449521231297 0.92459149094421322 -
0.57111146479995767 1.2737856648803554
0.26215195645915212;1.0659818039153526 0.54357631955169372 -
0.78070886491405 -1.1840788713561763 0.2693793062470165
0.65772955436717628 -0.26231518129102821 -1.1582883015760512;-
0.97681746052878926 -0.26540413163193038 -0.30116894339045042 -
0.086801336183695246 0.58953329871495141 0.04811683636383083
0.97646080048154804 0.37586259384355475;-0.75350203200996646
0.42677857628748667 -0.0048924820685517789 -0.20086490368901397 -
0.55985219707750555 -0.27427085762788145 0.56826046452935142
1.5454212219166441;-0.14244920909491576 0.74689499261852799
0.39068345349043332 -2.0964145665576721 0.95169093726424703
0.36653461602416515 0.60122236676686625 -0.61140293405554214;-
0.25287097541156683 2.069283751751251 1.7760725965142297
0.099328444819960296 1.9481923980030458 0.2901787277436701
0.92399044166188715 -0.83232004634660683;-0.31467552902030554
0.77773418233641778 0.056592938988008261 1.4142555041779137 -
0.30731452434226325 0.015364933561313254 -0.7609654126969545
1.2940579730047996;0.89585129156114252 -1.1153255057289291 -
1.5108271297246025 -0.14811032624220699 0.40756931093604332
0.67468356728972656 -0.040004488119369269 0.6522473699596808;-
0.60745234118871427 0.78993671675931409 -0.61111270902876913
1.2453742704933148 0.92552148860842121 1.0921452083844907
1.2654781598913636 -0.91426206246054065;-0.69757331846773674
1.0316849654637994 -0.48146770340011558 -0.068334340094118012 -
0.66011442087991579 1.1425573028005118 0.56639862255468254 -
0.48954445556379028;-0.68433975929024371 0.3898738806579316 -
0.93091249265002596 -0.94002653215017473 0.97256852033404018
1.5418385072548728 -0.13337634683378977 -
1.1565754961738481;0.4609130395353615 0.75792903657326516
1.2862998374837116 0.59086032152726742 0.98302961587895765 -
0.85845400217944556 -0.66943866696636589 -
0.36013374734911768;1.5694970477095167 0.27977116255715478
2.0983523297868132 -0.17133305109134528 1.3637678658234025 -
0.74229385337184606 0.64285461943966604 0.40335827054814266;-
1.7004002662537832 -0.53683464056175967 -0.89647770085279543
0.38881491138607338 0.86501068541299209 0.36878141428694644
0.21679952547822567 -0.91581060827020988;0.051623815239314072 -
0.93050198682215923 1.0119141072675084 -0.51267073459265777
1.0596305854948112 -0.26764669175213007 -0.66644978438436364
0.31899109649431917;-0.53456272617240541 -0.2812999088955212
1.4973065468758031 -0.94989165744377269 0.92989113077869023 -
1.367465421025587 1.5845118221603938 -1.3750569304797429;-
1.4035840642909994 0.46109647770584522 -0.098292478211244261
0.56298186383387661 -0.14393770983302073 -1.4966310605038564
0.57721641161006643 -1.295061898387075;-0.18333740824362635
0.58693660670489034 2.1244395396730282 1.0184438474477593 -
1.0491946686491382 -0.59615482497195338 1.3746469177066063 -
2.3737817076372227;0.62751398080673526 -0.96666351412068141 -
0.57702437231426429 0.23514696770468344 1.1886992731361949
0.13177550940830851 -1.9336528059008091
1.7607821120185703;1.2643126716992565 0.29211182710491113

```
0.41384418273350526 -0.016326296153362469 0.87378397651039974 -
0.4491508463141059 0.51319843614687477 1.7605042261089232];

% Layer 2
b2 = -0.16136644951890614;
LW2_1 = [0.51207571125722584 -0.69796620686533273 -0.7043045796002243
0.10080310389214667 -0.42215149105487876 1.2058716778961602 -
0.057402672828765343 1.0093594574518967 -0.34496419447174287
1.4145308201552578 0.5876537299784107 0.74790026161987666
0.25581001812294019 -1.6262088022239276 0.78782765469175708 -
1.6549546613048218 -0.90233365100231344 0.56460416780152545
0.6814495467997671 -1.2365938642320062 -0.53941996377332602 -
0.50385618176478453 -0.27349171364937302 -1.0818592928137074
0.40958643146634571 0.060644416022302458 -0.07706192455331852
0.85080857661178955 1.8291784292297053 1.7579837547443857 -
0.083077499051731674 -0.74732698493281402 0.44180543807831374 -
0.30071701878419105 0.56738198758846747 -0.88480669331213546 -
1.0370550277784769 -1.1179482075263121 0.94006063152767971 -
0.039248551767437231 -0.43791298035027132 0.12260245503261299
0.57417661773629192 -0.94697346965626483 -0.17229549180917364
0.35872519630936872 -0.95698829059821022 -1.5591726792257536
0.41594278809541574 -0.97243193830564523];

% Output 1
y1_step1.ymin = -1;
y1_step1.gain = 0.0480042243717447;
y1_step1.xoffset = 0.009;

% ===== SIMULATION ========

% Format Input Arguments
isCellX = iscell(X);
if ~isCellX
    X = {X};
end
if (nargin < 2), error('Initial input states Xi argument needed.'); end

% Dimensions
TS = size(X,2); % timesteps
if ~isempty(X)
    Q = size(X{1},2); % samples/series
elseif ~isempty(Xi)
    Q = size(Xi{1},2);
else
    Q = 0;
end

% Input 1 Delay States
Xd1 = cell(1,3);
for ts=1:2
    Xd1{ts} = mapminmax_apply(Xi{1,ts},x1_step1);
end

% Allocate Outputs
Y = cell(1,TS);

% Time loop
```

```
for ts=1:TS

    % Rotating delay state position
    xdts = mod(ts+1,3)+1;

    % Input 1
    Xd1{xdts} = mapminmax_apply(X{1,ts},x1_step1);

    % Layer 1
    tapdelay1 = cat(1,Xd1{mod(xdts-[1 2]-1,3)+1});
    a1 = tansig_apply(repmat(b1,1,Q) + IW1_1*tapdelay1);

    % Layer 2
    a2 = repmat(b2,1,Q) + LW2_1*a1;

    % Output 1
    Y{1,ts} = mapminmax_reverse(a2,y1_step1);
end

% Final Delay States
finalxts = TS+(1: 2);
xits = finalxts(finalxts<=2);
xts = finalxts(finalxts>2)-2;
Xf = [Xi(:,xits) X(:,xts)];
Af = cell(2,0);

% Format Output Arguments
if ~isCellX
    Y = cell2mat(Y);
end
end

% ===== MODULE FUNCTIONS ========

% Map Minimum and Maximum Input Processing Function
function y = mapminmax_apply(x,settings)
y = bsxfun(@minus,x,settings.xoffset);
y = bsxfun(@times,y,settings.gain);
y = bsxfun(@plus,y,settings.ymin);
end

% Sigmoid Symmetric Transfer Function
function a = tansig_apply(n,~)
a = 2 ./ (1 + exp(-2*n)) - 1;
end

% Map Minimum and Maximum Output Reverse-Processing Function
function x = mapminmax_reverse(y,settings)
x = bsxfun(@minus,y,settings.ymin);
x = bsxfun(@rdivide,x,settings.gain);
x = bsxfun(@plus,x,settings.xoffset);
end
```
