# Supplementary material for: Reconstructed data of landings for the artisanal beach seine fishery in the marine-coastal area of Taganga, Colombian Caribbean Sea
Source: Data Brief. 2020 Apr 22;30:105604. doi: 10.1016/j.dib.2020.105604 (PMC7200242; doi:10.1016/j.dib.2020.105604)
Supplement: Supplementary file 5 [file mmc5.pdf]

	SISTEMA DE INFORMACIÓN DEL SERVICIO ESTADÍSTICO PESQUERO COLOMBIANO- SEPEC	*F-INPV-*
	**CAPTURE AND FISHING EFFORT FORM**	**VERSIÓN: 02**

LOCATION OF THE REGISTRY

N°de registry (1)		Date (2)				Collector name (3)		Municipality (4)	
Landing site (5)		Fishing area (6)		Reference coastal site (7)					
Grid (8)		Depth in m (9)							

FISHING UNIT AND EFFORT INFORMATION

Fisher name (10)							Boat name (11)						Type of boat (12)
Propulsion method (13)							Number of fishermen (14)	Departure date (15)				Departure time (16)		
R	R y	P y R	Mot int	HP	Mot FB	HP		Arrival date (17)				Arrival time (18)		

CHARACTERISTICS OF THE FISHING GEAR AND / OR FISHING METHOD (19)

Gillnetting ( )							Longline ( )				Hand line ( )					Cast Net ( )		Net ( )			
Length (m)			Method				Quantity. hook		N° Throw		Quantity. hook					TM (Inch)	N°	TM cod-end (Inch)	Cam	Velao	Jal
Height. (N° net)			fixed	Ronza	Zang.	Cam	Type -Gauge		Method		Type -hook		Method					Purse seine (Ruche)			
Size (Inch)	Min	Max	Bol.	Lang.	Lang	Boq	Min	Max	pelag	bottom	Min	Max	fixed	Con	Con	height (radio)		Long. (m)		TM cod-end (inch)	

Artisanal trawlnet		Marucha ( )		Cóngolo ( )		Arrow ( )		Pots or traps( )					diving/ manual harvesting							
TM cod-end (inchs)		quantity		quantity		quantity		Langost.	Jaiba	Peces	quantity		Peces	Piangua	Caracol	Ostra	Chipichip	Lang	Cal	Mixto

INFORMATION OF THE LANDING CATCH

Species	State	Category	N° Ind.	Weight (kg)	Value	Species	State	Category	N° Ind.	Weight (kg)	Value

COSTS OF FISHING TRIP
Fuel
rental Gear
Ice
Provisioning
Bait
Others
Total costs
Total weight (kg)

Observations:
